# Organic Kainate Single Crystals for Second-Harmonic and Broadband THz Generation

**DOI:** 10.1021/acsami.2c18454

**Published:** 2023-02-02

**Authors:** Hani Barhum, Cormac McDonnell, Tmiron Alon, Raheel Hammad, Mohammed Attrash, Tal Ellenbogen, Pavel Ginzburg

**Affiliations:** † Department of Physical Electronics, Tel Aviv University, Ramat Aviv, Tel Aviv69978, Israel; ‡ The Center for Light-Matter Interaction, Tel Aviv University, Tel Aviv69978, Israel; § Tata Institute of Fundamental Research, Sy No 36/P Serilingampally Mandal, Hyderabad, Telangana500046, India; ∥ Schulich Faculty of Chemistry, Technion - Israel Institute of Technology, Haifa32000, Israel; ¶ Triangle Regional Research and Development Center, Kfar Qara’3007500, Israel

**Keywords:** zwitterionic crystal, hyperpolarizability, second-harmonic generation, THz, nonlinear organic crystal

## Abstract

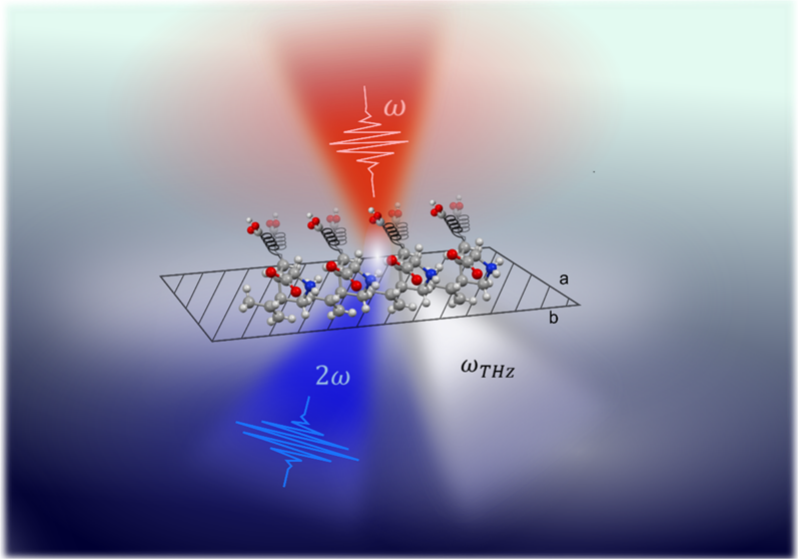

Organic crystals with unique nonlinear optical properties have been attracting attention owing to their capability to outperform their conventional nonorganic counterparts. Since nonlinear material responses are linked to a crystal’s internal microscopic structure, molecular engineering of maximally unharmonic quantum potentials can boost macromolecular susceptibilities. Here, large-scale kainic acid (kainate) single crystals were synthesized, and their linear and nonlinear optical properties were studied in a broad spectral range, spanning the visible to THz spectral regions. The non-centrosymmetric zwitterionic crystallization, molecular structure, and intermolecular arrangement were found to act as additive donor–acceptor domains, enhancing the efficiency of the intrinsic second-order optical nonlinearity of this pure enantiomeric crystal. Molecular simulations and experimental analysis were performed to retrieve the crystals’ properties. The crystals were predicted and found to have good transparency in a broad spectral range from the UV to the infrared (0.2–20 μm). Second-harmonic generation was measured for ultrashort pumping wavelengths between 800 and 2400 nm, showing an enhanced response around 600 nm. Broadband THz generation was demonstrated with a detection limited bandwidth of >8 THz along with emission efficiencies comparable to and prevailing those of commercial ZnTe crystals. The broadband nonlinear response and high transparency make kainate crystals extremely attractive for realizing a range of nonlinear optical devices.

## 1 Introduction

The nonlinear optical (NLO) properties of materials have attracted enormous attention since the development of laser sources and the subsequent observation of second-harmonic generation (SHG) among other nonlinear processes.^1,2^ Since then, the development and engineering of crystals with large second-order nonlinearities have attracted consistent research interest.^3−11^ While a range of nonlinear crystals including ZnTe, GaP, and lithium niobate are now in widespread use, organic structures have recently started attracting attention. Nonlinear frequency generation has been demonstrated in bulk crystals,^12^ molecular monolayers,^11,13^ and in single molecules,^14^ with a wide range of resultant optical phenomena such as SHG,^11,15,16^ broadband THz generation,^10,17−19^ multiphoton imaging,^10,16^ and quantum optics.^20^

In organic crystals, donor−π-acceptor molecular engineering is widely used to maximize the NLO response.^21^ This concept is based on tailoring pairs of electron donor and acceptor groups, linked through conjugated π-bonds as a charge bridge. This enables an efficient charge transfer between donor–acceptor units within a dipolar molecule. Non-centrosymmetric groups (though with some exceptions,^11^ also including nanostructuring^22−27^) are responsible for efficient SHG and THz generation. However, high intrinsic molecular hyperpolarizability β_*ijk*_ does not guarantee a macroscopic buildup of the nonlinear susceptibility χ_*ijk*_^(2)^, as a crystalline structure can average microscopic polarizabilities to virtually zero.^28,29^ Apart from strong intrinsic nonlinearities, efficient NLO materials should possess another set of properties, including low linear absorption, high environmental stability, high optical damage threshold, and fabrication reproducibility amongst others. For example, accommodating broadband nonlinearity and broadband transparency within the same crystal remains a challenge, which is the motivation for developing new material platforms.

The ability to design, simulate, and synthesize organic compounds with tailored optical properties has placed organic NLO (ONLO) materials as one of most investigated platforms in the field in recent years. In organic molecular design, numerical simulations are used in conjunction with chemical synthesis to enhance optical nonlinearity.^30−32^ Efficient frequency doubling, THz generation, electro-optic modulation, and high-speed integrated optics have been demonstrated with recently synthesized crystals and polymer layers such as DAST,^29,33^ OH1,^28^ and BNA.^7,8^ Flexible tailoring of their nonlinear response was achieved by introducing specific molecular changes within the crystal.^34^ The current main limitations of organic crystals include a narrowband transparency window, crystallization difficulties, and mechanical stability. To overcome these limitations, extensive investigations into new unique organic crystals have been undertaken.^8,29^ It has been found that crystallization of pure enantiomeric molecules leads to the formation of non-centrosymmetric crystals.^35^ From this structure, the crystal should have inherent second-order optical nonlinearity. Subsequently, we investigated for the first time the NLO properties of kainate crystals, synthesized from pure enantiomeric kainic acid. Kainic acid is a naturally occurring compound found naturally in some seaweed. These glutamic acid-based kainoids are known for being excitatory molecules in the central nervous system.^36−38^ In mild concentrations, these molecules stimulate neural excitatory signals and thus are used as a stimulating agent in the well-known epilepsy model.^39^ At high concentrations, kainate causes a continuous activation, followed by neural ablation of cells, which causes cell death. Nowadays, this acid is mostly used in biology, specifically as a neurotoxin^40^ or as a module for neurostimulation. The optical and physical properties of this material have yet to be determined.

Here, we develop controllable crystallization techniques for the growth of centimeter-sized kainate crystals and study their optical properties. First, the optical properties of the synthesized kainate crystals are fully developed using a combination of experimental and computational techniques, which show the high applicability of the crystal to nonlinear applications. This is revealed through broadband optical transparency, high quadratic optical nonlinearity, and highly effective molecular and crystal packing. Finally, the nonlinear applications are fully demonstrated for both SHG and THz generation across a broad pump spectral range.

## 2 Results and Discussion

### 2.1 Structural Characterization

**Figure 1:**
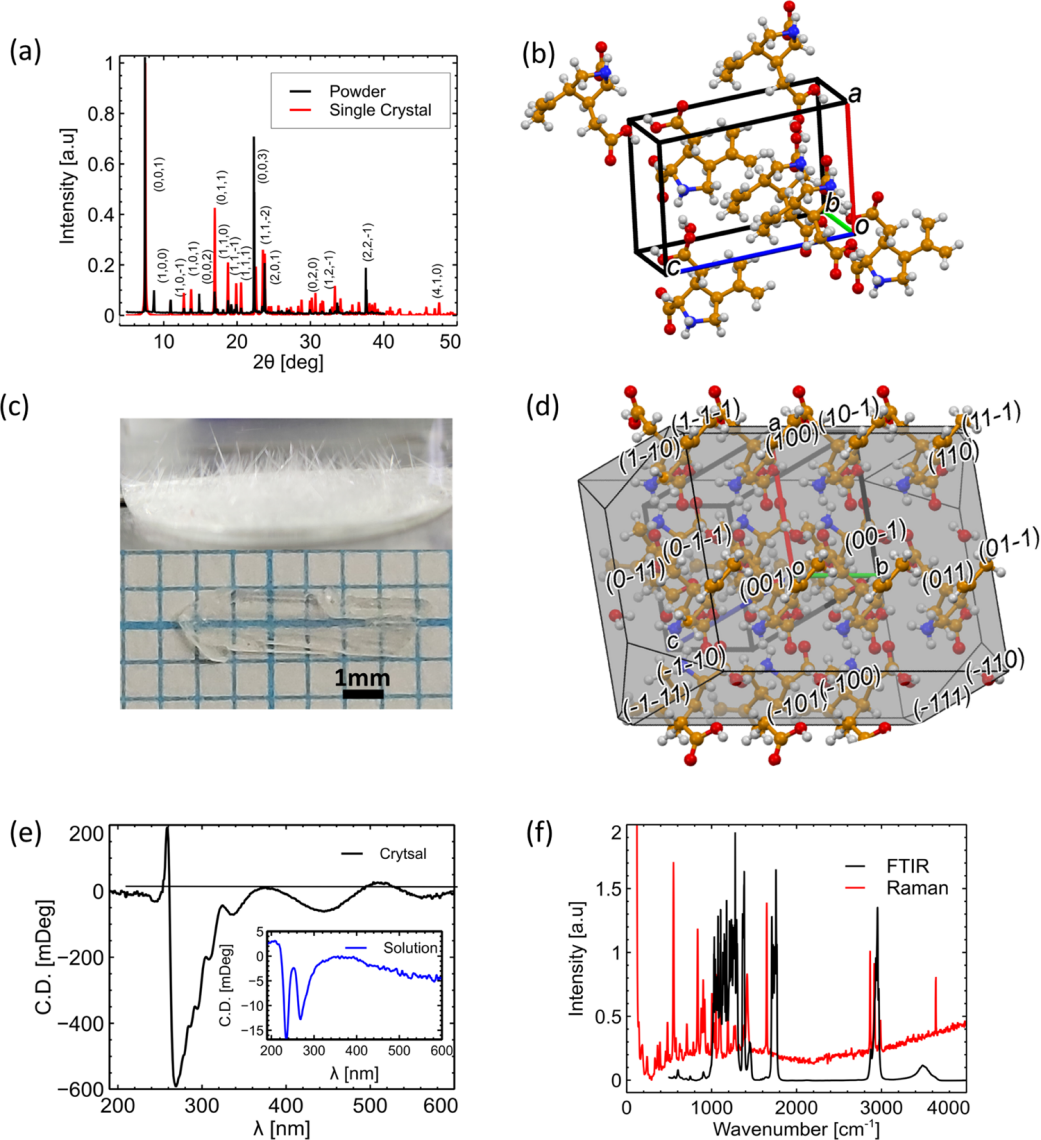
(a) Normalized XRD spectrum of a kainate crystal powder (black) and single crystal (red) with Miller indices (*h*,*k*,*l*). (b) Symmetric unit cell showing the repeated crystal structure. Gray spheres, carbon; red, oxygen; purple, nitrogen; and white, hydrogen. The cell axes are *a*, red (*x*); *b*, green (y); and *c*, blue (*z*). (c) Photo of crystals collected from the crystallization plate. Lower inset: a single kainate crystal. (d) Morphology of the crystal: several adjacent unit cells, as calculated from Mercury. (e) Circular dichroism (CD) spectra of the crystal. Inset: CD spectra of kainate in deionized water. A notable chirality of the crystal also appears in the blue optical region, while the molecule has only UV asymmetry. (f) Black curve: FTIR absorbance spectrum of kainate and red curve: Raman spectroscopy under 532 nm laser Nd:YAG excitation. The data sets are in arbitrary units to visualize the similarity of the molecular signatures.

Kainate molecules are chiral with kainate crystals, which were synthesized (see Supporting Information S1 and Section 4), and their structure was characterized using several techniques including X-ray diffraction (XRD), Fourier transform IR spectroscopy (FTIR), and Raman spectroscopy. Additional details on each technique are provided in Section 4. Figure 1a shows the overlaid XRD patterns of a single kainate monohydrate crystal and its ground powder. The overlap between the powder and single-crystal XRD patterns confirms the structure of kainate. Furthermore, these data were cross-correlated with the Cambridge Crystallographic Data Centre (CCDC) database.^41^ The data presented here confirm the zwitterionic structure, with a positively charged nitrogen and the negative neighboring carboxylic acid, both on a single kainate molecule. This feature plays an important role in crystal molecular orientations. l-Valine was added to the crystallization process to produce higher-quality crystals and was subsequently not detected in the XRD data. Other crystallization precursors could also be considered for this synthesis. Using the single-crystal XRD data, the Miller indices were obtained using Mercury software. The crystal axes *a*, *b*, and *c* were aligned with the *x*, *y*, and *z* polarizations, respectively. The symmetric unit cell parameters, crystal packing, and predicted crystal morphology were calculated in Mercury software^41^ (see Section 4) using the XRD data shown in Figure 1a. Figure 1b shows a visualization of the symmetric unit cell obtained from the collected XRD data.

Typical dimensions of the macroscopic crystal ranged between 1 and 2 mm in width and 1 and 2 cm in length. The average thickness was measured to be between 0.3 and 0.5 mm, with a typical crystal shown in Figure 1c. Visualization of the crystal growth at an early stage appears in Figure 1c. The crystals are elongated along the *b-*axis, which also corresponds to the direction predicted by Bravais, Friedel, Donnay, and Harker’s (BFDH) theory. The fast growth along this specific axis corresponds to the intermolecular forces governed by the permanent molecular dipole moment. This dipole–dipole interaction plays a crucial role in crystal formation. Participation of multipolar and chiral molecules results in an overall microscopic asymmetry of the structure. Figure 1d shows the (BFDH) crystal morphology calculated in Mercury.^42,43^ The unit cell axes in Figure 1b,c,d are aligned with the predicted morphology of repeated unit cells, shown in Figure 1d. All crystallographic data can be found in the cryptographic info file (CIF) found in the Supporting Information.

According to the XRD data, the crystal belongs to one of the Sohncke space groups that are non-centrosymmetric.^35^ In particular, the presence of the *P*2_1_ space group leads to crystalline chirality. The fraction of chiral molecules that crystallize into an asymmetric structure was found to be 18%.^35^ The *P*2_1_ group allows only symmetry operations of the first kind, i.e., rotation and translation. In our case, the polar axis is along the *y-*axis, which is aligned with the dipole moment of the unit cell. The dipole moment was calculated using a molecular dynamics simulation in MOPAC code^44^ and is oriented at θ_μ*i*_ = 3.2, 22.3, and 22.5° with respect to the *x*, *y*, and *z* axes, respectively. The addition of a water molecule aligns the dipole moment of the structure along the *y*-axis, meaning that the molecular main charge transfer axis is aligned parallel to the *b*-axis. The chirality was further examined using circular dichroism (CD) spectroscopy, and the results are shown in Figure 1e. The CD of the molecular solution of kainate, (2*S*,3*S*,4*S*)-3-(carboxymethyl)-4-(prop-1-en-2-yl)pyrrolidine-2-carboxylic acid, shows two peaks at 280 nm and 240 nm. The 240 nm peak corresponds to a transition related to the C=C double bond, while the 280 nm peak is related to the carboxyl groups. Small peaks at 350, 450, and 550 nm are related to the chirality inherited from the crystallinity packing. The solution of kainate causes a red shift of the spectrum peaks. The single crystal shows similar chirality relative to the solution, with more pronounced visibility in the difference between the excited states. The crystal has a main peak at 280 nm with several shoulders related to lower-intensity excited states. Figure S1 shows a mirrored crystal, where four water molecules from the crystal region are added. This scheme underlines the chirality, as the two crystals cannot be superimposed.

The FTIR and Raman shift molecular signatures of kainate in Figure 1f show a peak around 3500 cm^–1^, which corresponds to the stretching of the amine −NH group on the pyrrolidine ring.^45^ Three close peaks in the Raman spectrum appear around 2950 cm^–1^ and are related to the −CH stretching of the aliphatic carbons on the ring and on the alkene side group. Carboxylic −C=O stretching and −OH in-plane deformation appears at 1750 and 1390 cm^–1^ respectively, while off-plane deformation is around 900 cm^–1^. Another −C=O mode appears around 570 and 720 cm^–1^. Scissoring and twisting/rocking of −CH_2_ appear at 1460 and 1290 cm^–1^, respectively. The ring modes appear between 800 and 1100 cm^–1^, and mainly symmetric breathing comes at 900 cm^–1^. Similar peaks appear in the Raman spectrum, though with minor shifts and splitting.

FTIR data of the crystal show broad IR windows of transmission at 3300–1660 and 1000–500 cm^–1^. The crystal operation window is broad in the near- to mid-IR. The full Raman spectrum shown in Figure S2 has broad windows up to wavenumbers of 70 cm^–1^, with absorption peaks in the region of 200–100 cm^–1^.

### 2.2 Linear Optical Properties

**Figure 2:**
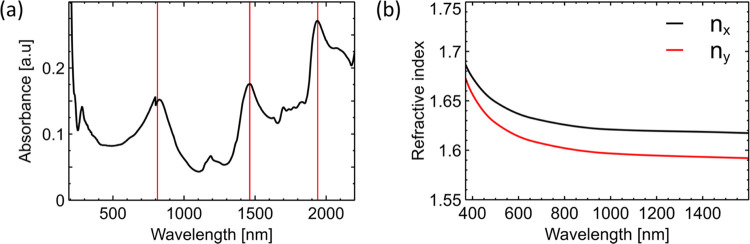
(a) Absolute absorbance spectrum of unpolarized light from a single kainate crystal; the red lines correspond to characteristic water absorption. (b) Refractive index dispersion calculated using the VASP simulation; the red and black solid lines correspond to the *x* and *y* components of the tensor.

The optical properties of the kainate crystals were measured experimentally and subsequently analyzed numerically. Absorbance in the UV–NIR region was studied using an integrating sphere to evaluate the absolute crystal absorption and is shown in Figure 2a. The absorbance values range from 0.02 to 0.3 mm^–1^ in the wavelength range of 200–2200 nm. Particularly, low absorbance is noted between 1000 and 1300 nm. The associated absorption coefficient calculated from the absorbance is shown in Figure S3a. Higher-absorbance regions can be seen at wavelengths of 900, 1500, and 2000 nm, related to characteristic NIR water absorption.^46^

Next, calculations of the crystal refractive index were performed in Vienna Ab initio Simulation Package (VASP).^47,48^ The simulation details can be found in Section 4. Figure 2b shows the corresponding refractive indices for both *x* and *y* crystal axes derived from the theoretical molecular dynamic simulations in VASP using the dielectric response in eqs 1 and 2. The refractive indices *n*_*x*_ and *n*_*y*_ have values of approximately 1.64 and 1.6 above ∼800 nm, respectively, showing the anisotropic properties of the crystal. At lower wavelengths, the refractive index begins to increase, with a rapid increase in values below 400–500 nm.

The experimental transmission of the crystals was measured using an OPO ultrashort laser system between wavelengths of 350 and 1600 nm and is shown in Figure S3b, demonstrating approximately 80–87% power transmission above 800 *nm*. The transmission wavelength trends and values are in the generally expected range, considering the refractive index values from the DFT simulations. Overall, the simulation shows slightly lower refractive index values compared to the experimental transmission data (simulation *n_x_* = 1.63 at 1000 nm, experimental *T* = 87% at 1000 nm, *n*_*x*_exp_ = 1.85). The difference between the experimental and simulated refractive index values is mainly due to the scattering of the transmitted light as it passes through the crystal, reducing the overall transmission. Defects inside the crystal volume and the crystal surface roughness are the main contributors to the reduced transmission,^49^ leading to a slightly overestimated refractive index. In the UV region, where a large increase in the refractive index is observed, losses occur due to material absorption bands, with the transition dipole strength calculated with time-dependent density functional theory (TDDFT) through CP2K code. The transition dipole is shown in Figure S3c, with the main bands at 225, 265, and 275 nm. The calculated transition dipole can be identified in the absorbance band and can be further verified in the CD spectra shown in Figure 1e. Another characteristic band appears at 355 nm, and it is responsible for generating optical blue fluorescence with a peak around 430 nm as shown in Figure S3d. The photoluminescence excitation spectrum is shown in Figure S3d, with the maximum intensity obtained at 360 nm.

### 2.3 Nonlinear Field Generation

#### 2.3.1 Structural Contribution to Nonlinear Effects

**Figure 3:**
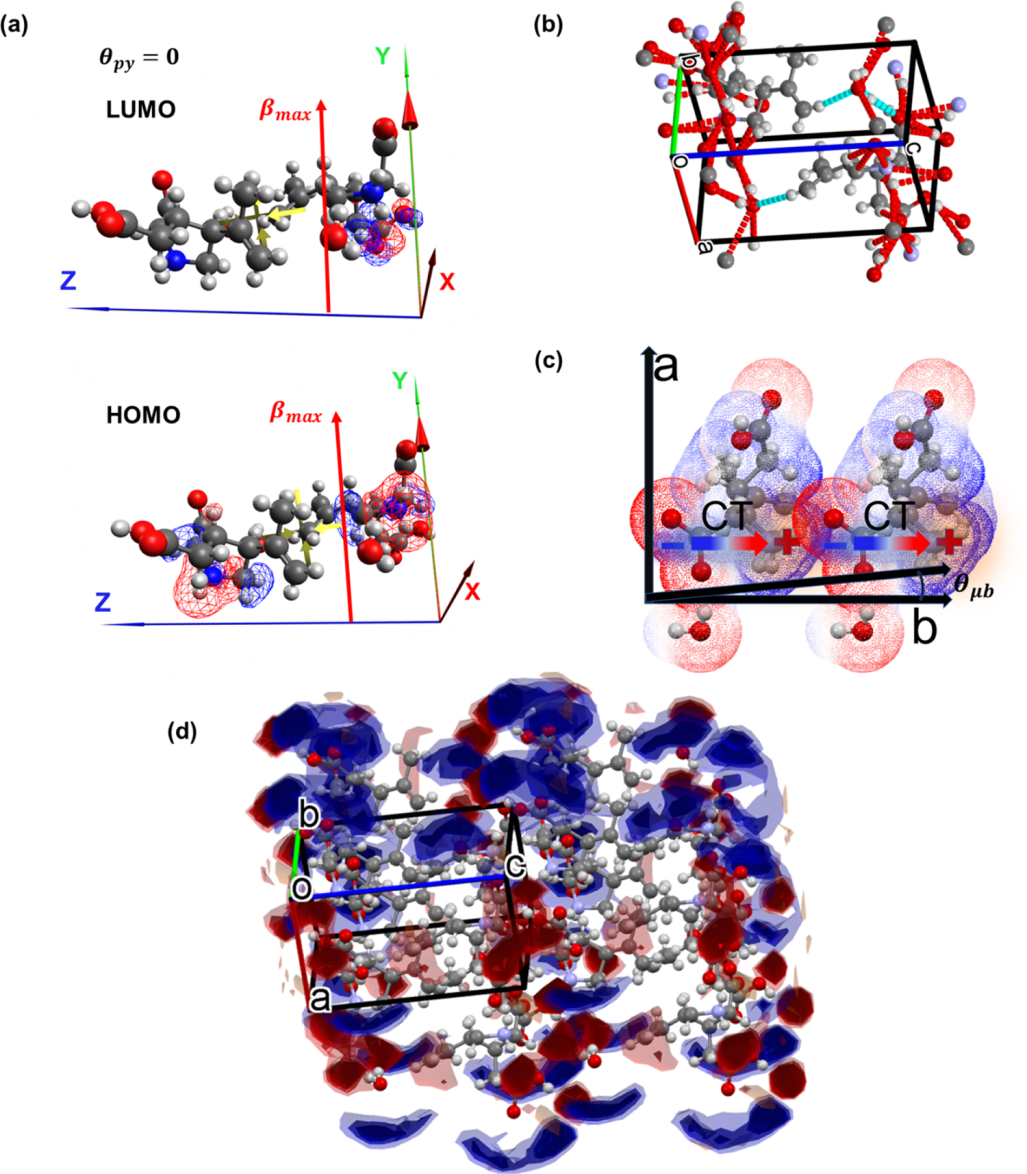
(a) Unit cell of kainate, consisting of two molecules with the defined crystal axes. The green arrow is the *y-*polar axis, and the red arrowhead represents the permanent dipole direction. The red arrow is the β_*yyy*_ direction, which happens to be the maximal hyperpolarizability. The molecular orbitals are drawn on the molecules as blue-red surfaces; the lower is the HOMO, and the upper is the LUMO. (b) Unit cell hydrogen and VDW interactions with its neighboring atoms. (c) Electrostatic potential map as calculated for an asymmetric unit cell aligned on the *x* and *y* crystal planes with the dipole moment direction and charge transfer axis shown. (d) Interaction map potentials. The blue surface is a negative charge, and the red surface is a positive charge.

Prior to the quadratic nonlinear effects observed from the crystal, the molecular orientation of the sample will be defined, as it plays a key role in the overall collective nonlinear response. Kainate molecules are packed in a *P*2_1_ group that has an eccentric structure; thus, the nonlinear coefficient does not vanish. This is similar to the DAST crystal, which has the same symmetry.^33^ The polar *y-*axis of the crystal is aligned perfectly parallel with respect to the permanent dipole moment, as illustrated in Figure 3a. Consequently, the order parameter is maximized for diagonal β_*yyy*_ ∝ ⟨cos θ*_kp_*⟩ and off-diagonal β*_yyy_* ∝ ⟨cos θ*_kp_*⟩ ⟨sin^2^ θ*_kp_*⟩ directions of the crystal, as described in eq 7a–7cc. The electron donor regions in a unit cell are mainly composed of the pyrrolic ring with the nitrogen atom; the acceptor part is the caboxylic part of the molecule. Furthermore, the simulations show that the highest occupied molecular orbital (HOMO) sets are on the pyrrolic, and the lowest unoccupied molecular orbital (LUMO) sets are on the carboxylic acid, as shown in Figure 3a. This supports the charge transfer (CT) direction proposed. From an intermolecular perspective, the net hydrogen and van der Waals (VDW) bonds that support CT along the polar axis are aligned with polar axes. Mainly, HO···HN and OOH···N are the bonds in the y-axis direction along with π-stacking forces, originating mainly from pyrrolic rings. The carboxylic hydrogen bonds are known for their strength; thus, they are more tightly bound^50^ with greater ability to CT. Figure 3b shows the intermolecular interaction of a unit cell, and Figure 3c shows an illustration of the head-to-tail orientation of the molecular dipoles. There are many diverse forces acting in the crystals plane. In the *x*–*y* plane, the molecules are aligned head to tail with shorter bonds along the *y* plane. In the *c*-axis, the molecules are aligned in a zig-zag form. Different crystal planes are shown separately in Figure S4a–c. The electrostatic potential of a unit cell calculated in MOPAC through Mercury software is presented in Figure 3c. The electrostatic potential of a unit cell with the crystal axis is presented in Figure S4d. The net electrostatic potential map of a symmetric unit cell, with the zwitterionic charged molecule aligned along the polar axis, is parallel to the charge transfer plane. Figure 3d shows that the crystal contains charged domains with symmetric alignment, suggesting a polymer-like behavior of the molecular chains. Support from the intermolecular interactions allows the whole structure to act as a unit, which generates a highly nonlinear response.

#### 2.3.2 Second-Harmonic Generation

**Figure 4:**
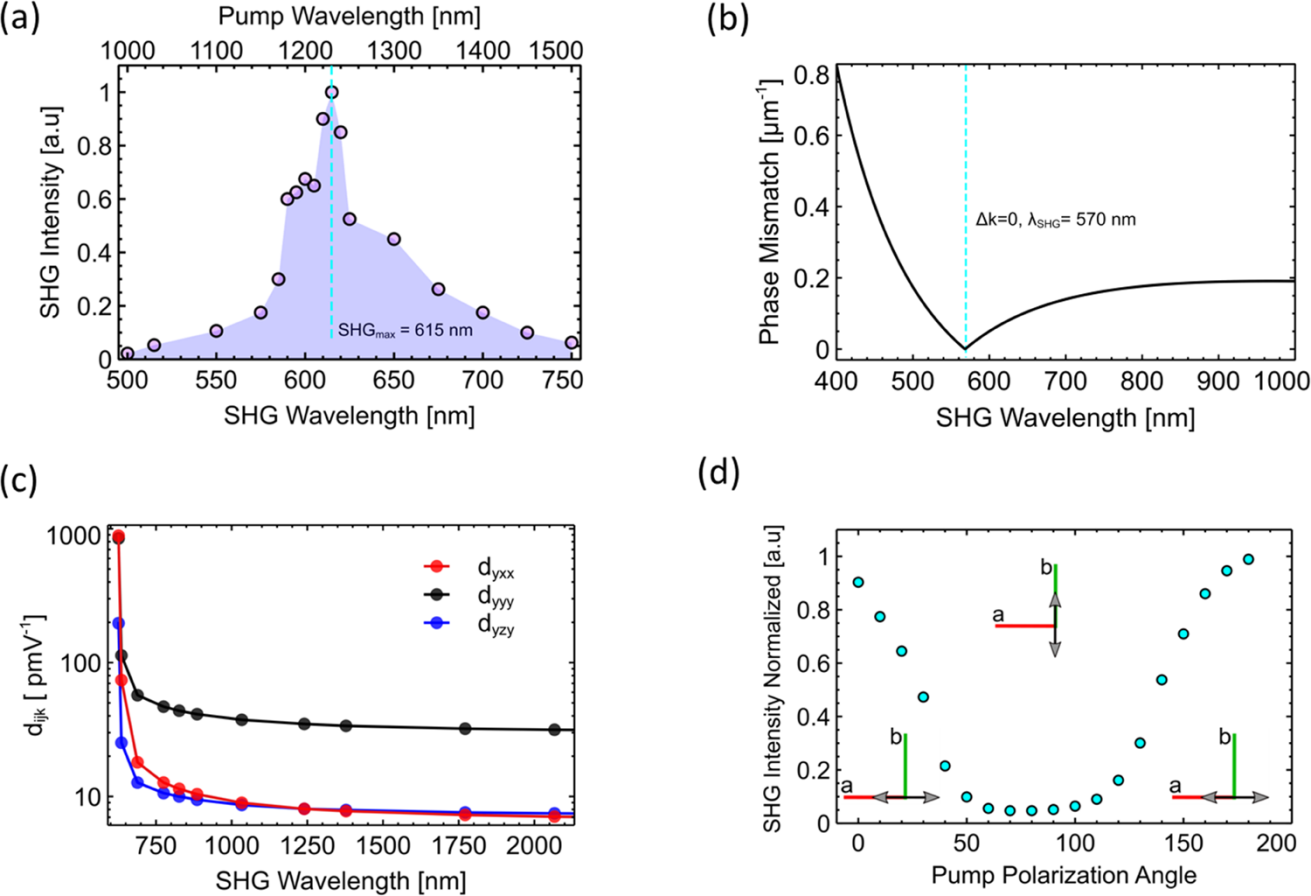
(a) Normalized second-harmonic generation from a kainate crystal using a range of ultrashort pump wavelengths from 1000–1500 nm. (b) Phase mismatch values, extracted from the simulated refractive index data, with the phase mismatch approaching zero at 570 nm. (c) Nonlinear optical susceptibility coefficients, calculated from the molecular hyperpolarizability. (d) Generated second-harmonic generation intensity as a function of pump linear polarization angle at a pumping wavelength of 1200 nm. The maximum generated signal occurs when the linear polarization angle is parallel to the crystal *x*-axis, with a minimum along the *y-*axis. The generated SHG polarization is always parallel to the crystal *y-*axis.

The nonlinear SHG properties of the kainate crystal were first examined as a function of the fundamental pumping wavelength using an OPO ultrashort laser system in the region of 1000–1500 nm (see Section 4). The measured SH signal as a function of wavelength is shown in Figure 4a. In this case, the crystal is orientated perpendicular with respect to the irradiating laser pulse with a linear polarization along the crystal *x*-axis. The SH shows a peak signal at 615 nm and falls off for both decreasing and increasing wavelengths, indicating an optimum phase-matching point at 615 nm. The simulated phase mismatch values between the pumping and SH beams were calculated from the simulated wavelength-dependent refractive index values from Figure 2b and is shown in Figure 4b. The phase mismatch approaches zero at an SH wavelength of 570 nm. This value is close to the experimental SHG maximum at 615 nm, with such a deviation within the limits of the DFT simulation accuracy. The effect of birefringent phase matching on the generated SH signal was also measured. Here, the crystal was angularly rotated around the *z*-axis with respect to the incident laser pulse, and the SH signal was optimized (see Supporting Information Figure S6). From this, the peak SH range could be extended, with enhanced emission observed at SH wavelengths between 400 and 700 nm.

The molecular hyperpolarizability was extracted in MOPAC under the (Moller–Pesset) MP6 approximation^51^ (see Supporting Information Section S6). Some of the NL coefficients of the crystal were estimated using the MOPAC molecular hyperpolarizability simulation using eqs 6 and7a and are shown in Figure 4c. Overall, the *P*2_1_ space group has 18 distinct second-order nonlinear coefficients, which are shown in Supporting Information S8. Here, we only show the main coefficients relevant to the study. The highest value is that of *d*_*yyy*_ over the entire spectrum, which is expected from a crystal with such symmetries. The theoretical values of *d*_*yxx*_ and *d*_*yyy*_ at wavelengths greater than 750 nm are approximately 10 and 40 pm V^–1^, shown as the red and black lines in Figure 4c, respectively. At lower wavelengths below 700 nm, the theoretical values of *d*_*yxx*_ and *d*_*yyy*_ approaches 200 and 600 pm V^–1^ at 650 nm, respectively.

From the direct measurement of the SH power output from the *x*–*y* plane, the *d*_*yxx*_ was calculated using eq 8a. The *d*_*yxx*_ in perfect phase matching at 570 nm is about 20 pm V^–1^ and agrees with the simulation results. Calculations at 600 and 550 nm are closer to 70 pm V^–1^. The calculations were obtained far from the phase-matching function zeros to prevent the effects of the function sensitivity in those regions. The damage threshold of the crystal was measured to be approximately 0.5 J·cm^–2^, which is in the range of organic crystals with a melting point of 528 K. The overall SHG average power conversion was experimentally measured using a sensitive power meter and appropriate filters at a pumping wavelength of 1200 nm, giving average values of ∼ 4%, with a normalized conversion of 10^–3^ % W^–1^ cm^–2^. The efficiency value is limited by the damage threshold of the crystal.

Finally, the effect of linear polarization angle on the generated SH was examined and is shown in Figure 4d. Rotating the pump beam linear polarization away from the crystal *x*-axis results in a decrease in the generated SH power, with a maximum value when the beam is polarized along the *x*-axis. The SH beam was filtered with a polarizer to verify its polarization direction. In this facet of the crystal, *d*_*yxx*_ is the active NL coefficient.

#### 2.3.3 Nonlinear THz Generation

**Figure 5:**
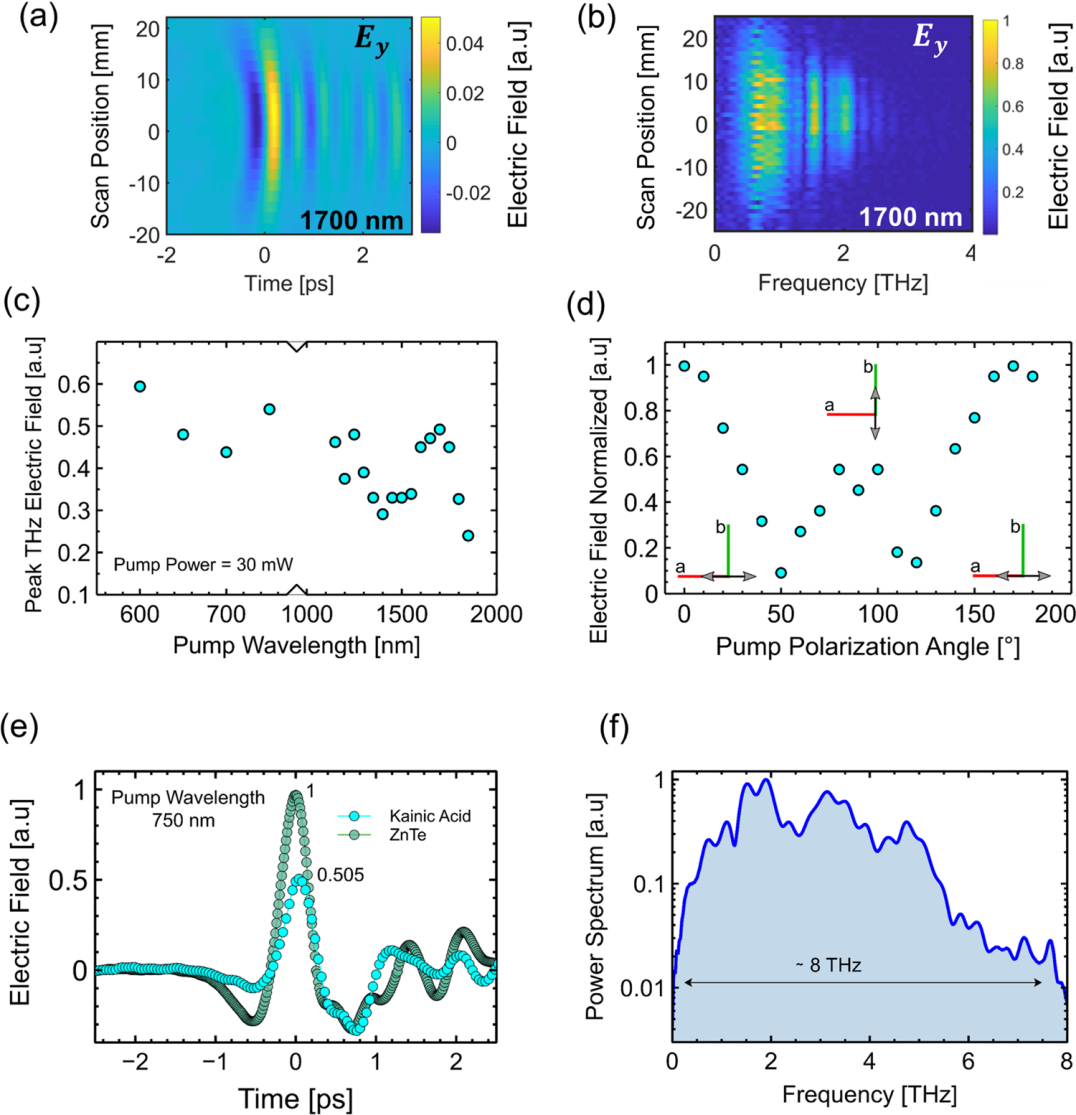
(a) Spatio-temporal properties of the generated THz pulse from the kainate crystal for a pumping wavelength of 1700 nm. The THz pulse has an approximate 0.5 ps FWHM pulse duration. (b) Spatio-frequency properties of the generated THz pulse; the frequency band extends up to 2 THz. (c) Peak THz electric field as a function of pumping wavelength from 600 to 1900 nm. An OPA laser and BBO nonlinear crystal were used to cover the wavelength range. (d) Generated peak THz signal as a function of the pump linear polarization angle; the maximum signal is observed when the linear polarization is parallel to the *x*-crystal axis. (e) Emitted THz electric field amplitude versus time for a ZnTe nonlinear crystal and a kainate crystal at a pumping wavelength of 750 nm and a power of 15 mW. The electric field of the kainate crystal is half that of the ZnTe. (f) Measured THz bandwidth from the kainate crystal using a thinner GaP detection crystal. The bandwidth is approximately 8 THz.

The nonlinear THz generation properties of kainate crystals were also examined. The kainate crystals were illuminated with two ranges of ultrashort pulses. The first range consisted of the output of an OPA laser, giving an IR wavelength band from 1150 to 1800 nm. The second pumping band was from 600 to 750 nm, which was obtained from the combination of the OPA laser and a BBO nonlinear crystal. The resulting THz electric fields were measured using time domain spectroscopy (see Section 4). Figure 5a,b shows the spatio-temporal and spatio-spectral properties of a generated THz pulse, after illumination with 1700 nm ultrashort pulses. The generated single-cycle THz pulse has a FWHM of approximately 0.5 ps, resulting in a broadband THz field up to 2.5 THz limited by the ZnTe detection crystal, with a linear polarization in the *y* direction. The effect of laser wavelength on the THz field amplitude is shown in Figure 5c. In general, the THz amplitude shows no real appreciable trend in the region of 1150–1750 nm; however, above this point, the amplitude begins to noticeably decrease. For pumping wavelengths in the visible region, an increase in the THz field amplitude is observed, with a maximum signal recorded at 600 nm. In general, the temporal and spectral properties are not affected by the pumping wavelength. The effect of the pumping linear polarization angle was also measured and is shown in Figure 5d. Here, the angle was rotated through around 180° at a fixed pumping wavelength of 1250 nm, and the associated THz electric field was measured. The highest electric field amplitudes were observed when the pumping angle was parallel to the *x*-crystal axis, with a minimum observed for an angle of 45 and 135° from the *x*-axis. A second peak is observed when the polarization is parallel to the *y-*crystal axis; however, the peak amplitude is approximately half compared to the amplitude of the *x*-axis. The efficiency of THz generation from the kainate crystal was compared to a commercial ZnTe nonlinear crystal (0.1 mm thickness), as shown in Figure 5e. At a pumping wavelength of 750 nm and an average laser power of 15 mW, the THz electric field ratio of ZnTe–kainate was 0.5. The THz bandwidth of the kainate crystal was further analyzed by measuring the spectrum using a thinner GaP crystal (*d*_GaP_ = 0.1 mm), and the resulting emitted spectrum is shown in Figure 5f. The bandwidth for this configuration approaches 8 THz. The refractive index of the kainate crystal in the THz spectral region was measured using time domain spectroscopy and a ZnTe nonlinear source (see Section 4). The refractive index was measured to be ∼2 at 1 THz.

The THz nonlinear coefficient *d*_THz_ is related to the electro-optic coefficient *r* through the equation h  where . From this, the theoretical value of the nonlinear coefficients from the MOPAC simulation is ≈100 pm V^–1^ for *d*_*yxx*_ around 700 nm, while the *d*_*yyy*_ for THz has a theoretical value of 200 pm V^–1^ but could not be measured in our current experimental setup, as this crystal component is not easily accessible.

## 3 Conclusions

A new highly nonlinear organic kainate crystal was synthesized and characterized. Molecular features of the di-ionic molecule with a donor−π-acceptor motive aligned along the main charge transfer axis, and molecular packing of the crystal creates a non-centrosymmetric nonlinear optical active structure. The intermolecular net of bonds generates an optimally aligned structure between the polar axis and the crystal axis. The high broad transparency in the UV–NIR band enabled a broad nonlinear activity. The charged groups along the polar axes with a proton that can oscillate along and a heterocyclic ring all that are summarized into strong nonlinear SHG and broadband THz generation. The THz emission is approximately as strong as a perfectly phase-matched ZnTe commercial crystal, however with a much broader emission bandwidth. This organic structure first introduced here can be further integrated in applied nonlinear materials. Furthermore, enhanced chemical engineering of the structure can be studied to generate higher nonlinearity and more stable derivatives of this structure.

## 4 Methods

### 4.1 Synthesis and Crystal Growth

Pure ((2*S*,3*S*,4*S*)-3-(carboxymethyl)-4-(prop-1-en-2-yl)pyrrolidine-2-carboxylic acid)(−)-kainic acid (99.8%) and l-valine 99.9% were purchased from Sigma-Aldrich Ltd. Those components were dissolved in deionized water (DIW 18 MΩ) and mixed with vortex for 30 min to ensure saturation. The solutions were filtered in 0.22 μm vacuum filters (Corning) and then poured into a 2 in. glass Petri dish.

The crystals start forming a small needle after several minutes; then, it is left to slowly grow for 48–72 h. To achieve 1–2 cm crystals, another growth step was done by placing samples into a saturated solution, followed by slow evaporation for another 72 h. The crystals were then placed on a fiber-free paper to dry before further characterizations and slightly polished.

### 4.2 X-ray Diffraction (XRD)

A Bruker Smart Apex Duo installation with a Cu Kα source and an Apex 2D detector were used for characterization of crystals. Diffraction patterns were measured at a 30° angle, formed by the detectors’ normal and the incident X-ray beam. The two-dimensional (2D) data were subsequently recalculated to the standard 2θ configuration. HRXRD was performed on carefully selected single crystals.

### 4.3 Fourier Transform IR (FTIR) Spectroscopy

FTIR measurements were performed with a Vertex 70 (Brucker’s) spectrophotometer. The crystal sample was placed on a mirror sample holder with a silicon background. The measurements were performed in the reflection mode. Data units were converted from transmittance to absorbance in the range of 1–20 μm. A 20° reflection angle was chosen.

### 4.4 Raman Spectroscopy

Raman spectroscopy was performed with an OlympusIX71 inverted microscope, equipped with a 50× objective. The output is coupled to a confocal spectrometer (Horiba LabRam HR). The sample was excited using a 532 nm laser with 5 mW average power. No cover glass was present between the crystal and objective during measurement to avoid distortion of the signal.

### 4.5 UV–VIS–NIR Spectroscopy

The optical absorption measurements were performed with an optical spectrophotometer UV–vis–NIR Cary 5000 (Varian, Agilent Technologies), covering the wavelength range of 170–2600 nm. For kainate measurements, an integrating sphere was used to collect the scattered light and estimate the total crystal’s absorption. The sample was placed on top of a silicon substrate and above a Teflon substrate. A reference signal was obtained for calibration.

### 4.6 Circular Dichroism (CD) Measurements

Circular dichroism (CD) spectroscopy was performed using a Chirascan CD spectrometer (Applied Photophysics, UK), working in the range of 190–600 nm. For CD measurements, the kainate solutions were placed in a quartz cuvette with a 2 mm pathway (Thorlabs) and measured directly. The crystals were mounted on a custom-made holder.

### 4.7 VASP Refractive Index Simulation

The experimental lattice cell parameters for the calculations were extracted from the XRD data. The structure was geometrically relaxed using projector-augmented wave (PAW) potentials with generalized gradient approximation (GGA-PBE).^52−54^ A cutoff energy criterion of 400 eV was used, while the electronic ground convergence criterion was set to 10^–4^ eV. Furthermore, the force convergence criterion was set to 10^–2^ eV Å^–1^ for geometry relaxation. The Brillouin zone (BZ) was sampled by a 3 × 4 × 2 Gamma centered grid point scheme. For computing the dielectric properties of the material, a large number of empty bands were taken. The number of bands used in the current study is 1.5* (number of ions + number of valence electrons present in the system). This is 3 times the default used by VASP.

The optical properties are calculated using the frequency-dependent complex dielectric function
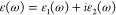
1where ω is the frequency of electromagnetic (EM) radiation.

The real part and the imaginary part are mutually related by the Kramers–Kroning (KK) relation. The complex refractive index of the form *N*(ω) = *n*(ω) + *ik*(ω) can be obtained using the dielectric function, where *n*(ω) and *k*(ω) are of the form
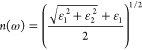
2
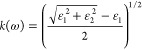
3

### 4.8 Nonlinear Optical Model

The macroscopic optical polarization of a bulk material under an applied electric field can be expressed in a Taylor series as^55,56^
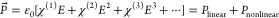
4where χ^(*n*)^ are tensorial nonlinear susceptibilities. The microscopic dipole moment, polarized by the external field is given by
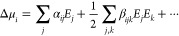
5where μ_*i*_ is *i*th Cartesian component of the dipole moment, α_*ij*_ is the linear polarizability, and β_*ijk*_ is the first-order hyperpolarizability, which is responsible for the second-order nonlinear properties.

An organic χ_*ijk*_^(2)^ can be defined from an oriented gas model^21^ as
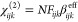
6where *N* is the molecular density, *F*_*ijk*_ are the *f*_local_, local field factor , *n*_*x*ω_ is the *x*-axis refractive index at frequency ω, and θ*_kp_* is the angle between the polar axes and crystal axes. The hyperpolarizability is given by
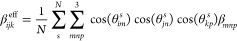
7awhere θ_*im*_^*s*^ is the angle between the crystal and molecular axes. Equation 7a underlines the impact of the crystalline structure on bulk NLO properties. The diagonal β_*iii*_^eff^ and off-diagonal β_*iij*_^eff^ hyperpolarizabilities are given as
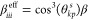
7b
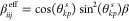
7cThe nonlinear coefficient *d_yxx_* was calculated from the experimental data around the phase-matching point at 570 nm. In the simplest case of the undepleted pump with a gaussian beam, the power at the crystal placed at *z* = 0
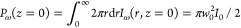
8awhere *w*_0_ is the beam waist, and *I*_0_ is the intensity. The frequency doubling effective coefficient is given by
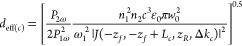
8bwhere .

*P* is the power, ω is the frequency, *n* is the refractive index, *z* is the crystal position, *k* is the wavenumber, *c* is the speed of light, and ε_0_ is the permittivity of vacuum.

### 4.9 SH Generation and Detection

The SH from the kainate crystal was generated with two different femtosecond laser sources. An optical parametric oscillator (OPO) laser (Coherent Chameleon) was used for pumping at wavelengths from 1000 to 1500 nm. The system generates 170 fs wavelength-tunable pulses at an 80 MHz repetition rate. A convex lens (*f* = 150 mm) was used for focusing onto the sample. A second microscope objective was used to collect the SH signal.

For NIR wavelengths between 1064 and 2500 nm and to investigate the effects of birefringent phase matching, a tunable femtosecond laser source (Spectra Physics Solsits Ace), generating 35 fs at 800 nm and 3.5 mJ per pulse, was used. The pulses are then directed into an optical parametric amplifier (OPA) (TOPAS) where the desired wavelength could be selected. The laser beam was focused onto the sample with a convex lens (*f* = 150 mm), resulting in an approximate spot size of 100 μm at 1200 nm wavelength. The kainate crystal was placed at the focal point of the lens on a multiaxis mechanical stage for controlling the spatial position and angular orientation of the sample. The SHG was collected using an off-axis parabolic mirror (*f* = 101 mm) and directed toward a spectrometer, which recorded the SH wavelength and intensity (Ocean Optics). The SH conversion efficiency was also measured directly using a low-power sensitive power meter, after appropriate filtering of the pump pulses.

### 4.10 THz Generation, Detection, and Refractive Index Measurement

THz generation was examined using an OPA ultrafast laser system. The optical system for detection was based on pump probe time domain spectroscopy. The pump beam of the OPA was split into two paths using a 99:1 beam splitter, where the 99% was directed into the OPA, and the 1% was used for the probe beam line. For THz generation, the kainate crystal was placed in the collimated beam of the pump laser, where telescopic optics were used to adjust the beam size for illuminating the full crystal’s facet. The pump beam was filtered out from the detection path using a thick Teflon slab, which only transmits THz pulses. For the OPA, output was used for pumping wavelengths between 1850 and 1150 nm. The pump wavelength was frequency-doubled using a commercial BBO crystal to obtain wavelengths between 600 and 800 nm. The generated THz field was collected and collimated using an off-axis parabolic mirror (*f* = 101 mm). In this collimated THz plane, a slit on a motorized stage was used to sample the spatial properties of the THz beam. A second off-axis parabolic mirror (*f* = 101 mm) was used to focus the THz beam into a ZnTe NLO crystal (*d* = 0.5 mm), which was used for electro-optic detection. To measure the extended THz bandwidth, the detection crystal was changed to a GaP NLO crystal with a thickness of 0.1 mm. To reduce THz absorption due to humidity in the air, the entire measurement setup was placed in an enclosure and pumped with dry air to a humidity of less than 1%. On the probe line, the probe beam was sent to a calibrated motorized stage, which was used to control the temporal delay. The probe beam was then directed through a hole in the second parabolic mirror and spatially and temporally overlapped with the THz pulse in the nonlinear crystal. The spatio-temporal electric field amplitude and phase was measured through the process of electro-optic detection, which is an optical set, containing a quarter wave plate, a Wollaston prism, and a balanced photodiode. The photodiode signal was amplified using a lock-in detector, which was synchronized to the laser source using a mechanical chopper. The refractive index of the kainate crystal was also measured using the same system. For this, a ZnTe nonlinear crystal was used as the source, which generates broadband THz fields up to 2.5 THz. The transmission of the generated field from the ZnTe through the kainate crystal was measured, as well as a reference through air. Total scan lengths of 100 *ps* were undertaken to obtain a high frequency resolution.

## Supporting Information Available

Material, crystal chirality, Raman shift spectroscopy, electronic band gap and optical losses, crystallographic planes, theory, computational details, and second-harmonic generation with birefringent phase matching (PDF)
Crystallographic data from XRD experimental data (CIF)

## Supplementary Material

am2c18454_si_001.pdf

am2c18454_si_002.cif
