# Supplementary material for: Organic Kainate Single Crystals for Second-Harmonic and Broadband THz Generation
Source: ACS Appl Mater Interfaces. 2023 Feb 2;15(6):8590–600. doi: 10.1021/acsami.2c18454 (PMC9940106; doi:10.1021/acsami.2c18454)
Supplement: Supplementary file 1 — am2c18454_si_001.pdf [file am2c18454_si_001.pdf]

Supporting Information

Organic Kainate Single Crystals for Second Harmonic and Broadband THz Generation

Hani Barhum[1,2,3,*,=], Cormac McDonnell[1,2,=], Tmiron Alon[1,2], Raheel Hammad[4], Mohammad Attrash[5] Tal Ellenbogen[1,2] and Pavel Ginzburg[1,2]

[1] Department of Physical Electronics, Tel Aviv University, Ramat Aviv, Tel Aviv 69978, Israel

[2] The Center for Light-Matter Interaction, Tel Aviv University, Tel Aviv, 69978, Israel

[3] Triangle Regional Research and Development Center, Kfar Qara' 3007500, Israel;

[4] Tata Institute of Fundamental Research-Hyderabad, Sy No 36/P Serilingampally Mandal, Telangana 500046, India

[5] Schulich Faculty of Chemistry, Technion – Israel Institute of Technology, Haifa 32000, Israel

= equal contribution

*Corresponding author – Hanibarhum@mail.tau.ac.il

S1. Materials

(-) – Kainic Acid 99.9%, L – Valine 99.99% was purchased from Merc group. DIW water was taken from an Ionic filter and the resistance of DIW was 18 MΩ. 0.22 µm Filters were purchased from Corning ltd.

S2. Crystal Chirality

Kainate molecules are chiral. The Kainate crystal belongs to the p_{21} space group that has no inversion symmetry. In this case, the crystal pack containing four water molecules was mirrored and no inversion symmetry can be seen in Figure S1.

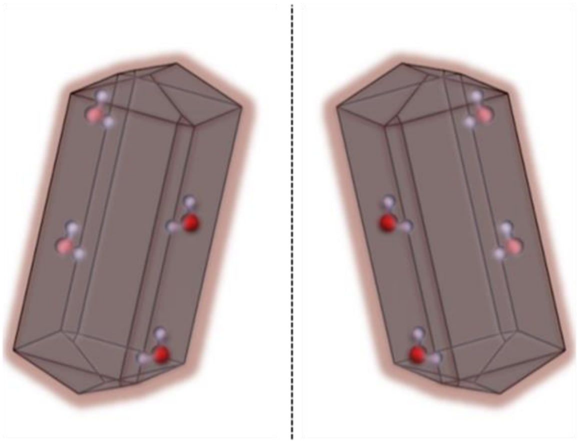

Figure S1. Two mirrored/enantiomorph crystals of Kainate. The crystals appear in the predicted BFDH morphology with the insertion of four water molecules. The crystal morphology can be symmetrically rotated in 180^0, but although the trimmed corners seem to be overlapping, the water molecules will be in a different position. Thus, there is no inversion symmetry, and the crystal packing is chiral.

S3. Raman Shift Spectroscopy

The spectrum obtained for 532 nm laser excitation shows different features that are due to radiation–crystal interaction and are shown in Figure S2. The higher wavenumber shift can be interpreted as due to the backbone stretching of the pyrrolic ring in a stack structure.

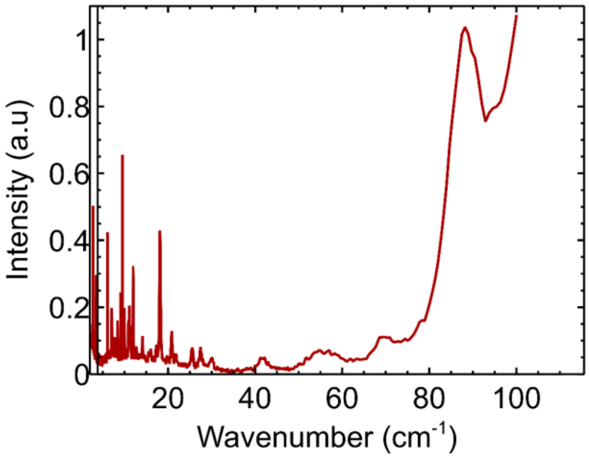

Figure S2. Raman Shift of Kainate crystals at 532 *nm* excitation pump. The lower wavenumbers appear in the main manuscript text. Higher wavenumbers in the crystal show the stretching of stacked rings.

S4. Electronic Band Gap and Optical Losses

The absorbance and the linear absorption coefficient were extracted from the absolute absorbance, measured using a Cary5000 spectrophotometer, and are shown in Figure S3 (a). An integrating sphere was used to measure the optical density of the crystal. The absorption coefficient of a 300 *µm* crystal was found to be $k = 2.303\, A \cdot d^{-1}$, where *k* is the absorption coefficient, *A* is the absorbance, and *d* is the crystal thickness. In Figure S3 (b) the transmission through the Kainate crystal is shown for *x*- and *y-polarized* light at wavelengths from 350 to 1600 nm, using an OPO ultrashort laser source. The transition dipole was simulated using CP2K code and was used to obtain the electronic transitions of Kainate shown in Figure S3 (c), which were correlated with the CD spectra. The calculated bandgap around 350 *nm* agrees with the maximum fluorescence measured, which is shown in Figure S3 (d).

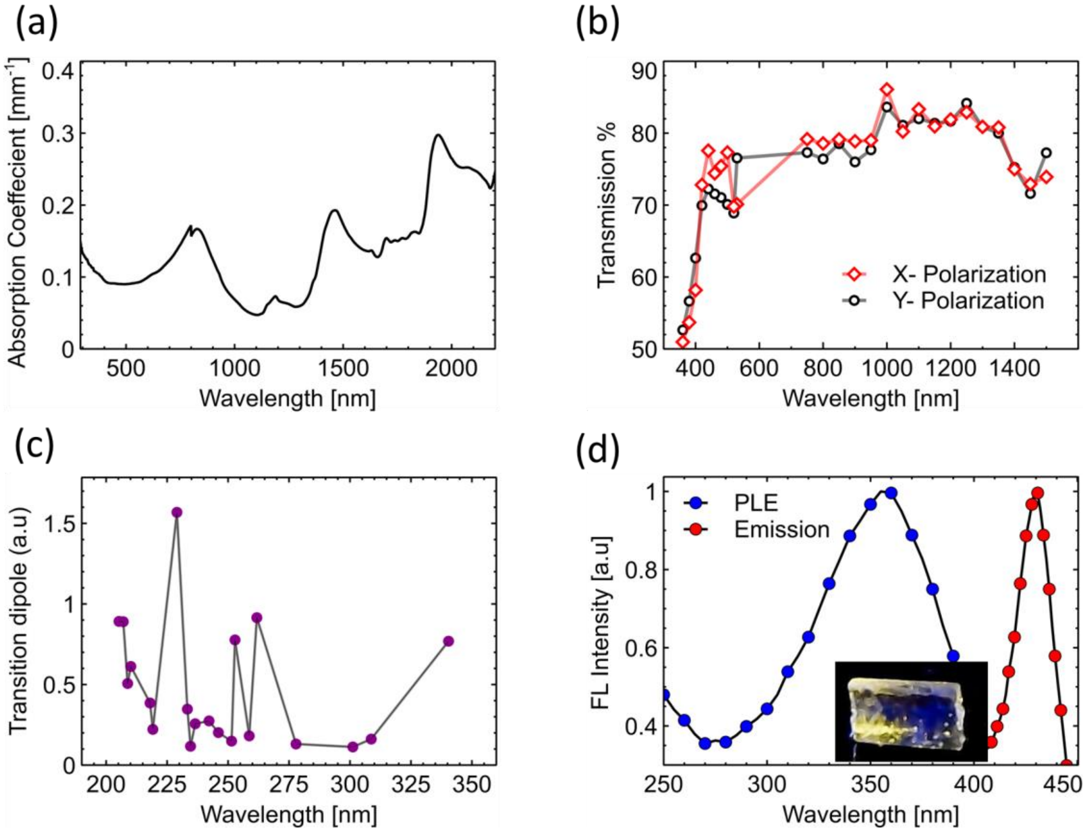

Figure S3. (a) Absorbance coefficient from absolute absorbance of the Kainate crystal after reflection in an integrating sphere between wavelengths of 300 - 2200 *nm*. (b) Experimental transmission of linear *x* and *y* polarizations through the Kainate crystal using an OPO ultrashort laser system. (c) Electronic transition dipole simulation (oscillator strength) between wavelengths of 200-350 *nm.* The data represents the relative strength of each electronic transition. (d) PLE spectra of Kainate crystal at 430 *nm* emission, with excitations in the range of 250-400 *nm* in 5 *nm* steps (blue dots). Fluorescence emission spectra of Kainate crystal (red dots). Inset - photo of a Kainite crystal illuminated with UV light and subsequent blue emission optical image.

S5. Crystallographic Planes

The crystal planes contain an ordered intramolecular bond. The bond map at each crystal plane explains the nonlinearity of the crystal. In organic crystals the Donor-π-Acceptor motive is one of the main features to obtain high nonlinearity, the crystal planes are plotted along with the electrostatic interactions. Noticeable charge transfer along the *y-axis* appears between the

charged groups. Also, a proton transfer process can occur on this axis from proton oscillations between the carboxyl and amine.

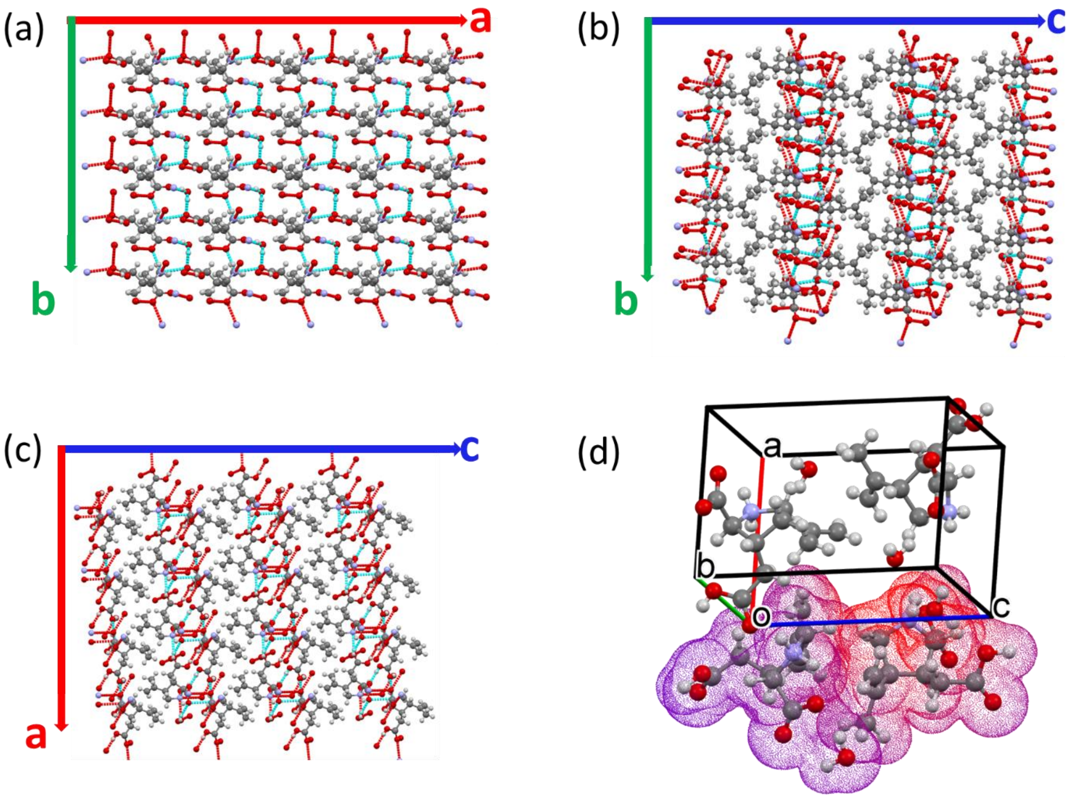

Figure S4. 2D sections of molecular crystal at different axes pairs. The red and light blue lines are the intermolecular interaction between neighboring molecules (a) a – b plane (b) b – c plane (c) c – a plane (d) two-unit cells, one with crystallographic axes (top) and another with electrostatic potential distribution as calculated from Mercury MOPAC in AM1 (down).

S6. Theory and Computational Details

The polarizability and hyperpolarizability of the crystal with respect to crystal volume were calculated using MOPAC in the PM6 approximation. The volume hyperpolarizability has a linear relationship in the simulation, suggesting an approximated periodic boundary condition for the molecular unit cell. This relationship is supported by the experimental results obtained for the refractive tensor. The data from the experiment and VASP DFT approach is less accurate.

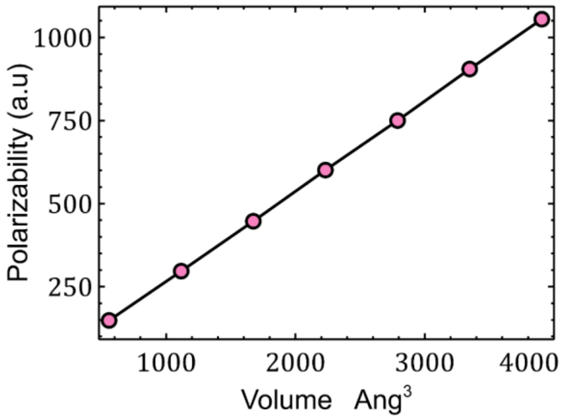

Figure S5. Averaged polarizability per unit volume for increasing values of crystal volume simulating a periodic boundary condition.

The refractive tensor was also calculated from the molecular polarizability extracted in MOPAC in (Moller - Pesset) MP6 approximation[1]. To replicate periodic boundary conditions for the crystal structure, a manual investigation of the polarizability was done for different and increasing numbers of unit cells in a finite-size crystal (e.g.[2],[3] for similar approaches). Once a linear relation between the crystal's volume and the macroscopic polarizability is obtained, the responses resemble those of an infinite crystal, shown in Figure S5. This semi-empirical calculation is limited in the prediction of molecular interactions mainly due to the neglection of overlapping integrals. The bulk refractive index tensor was retrieved from the Clausius-Musette relation[4] as shown in the following relation:

$$\frac{n_{ii}^2 - 1}{n_{ii}^2 + 2} = \frac{N \cdot \alpha_{ii}}{3\varepsilon_0 V} \tag{s1}$$

where n_{ii} is the diagonal refractive index element, NV^{-1} is the molecular density ($N = 2,\ V = 5.58 \cdot 10^{-32} m^3$)), ε_0 is the vacuum permittivity, and α_{ii} is the polarizability component. Thus, the diagonal refractive tensor of Kainite can be calculated with the above relationship between the molecular polarizability and refractive index.

S7. Second Harmonic Generation with Birefringent Phase Matching

The effect of angular rotation of the crystal relative to the pumping pulse on the SH properties was examined and is shown in Figure S6. Here the magnitude and bandwidth of the generated SH can be expanded, with the peak SH values expanding out to SH values of 450 -500 *nm*.

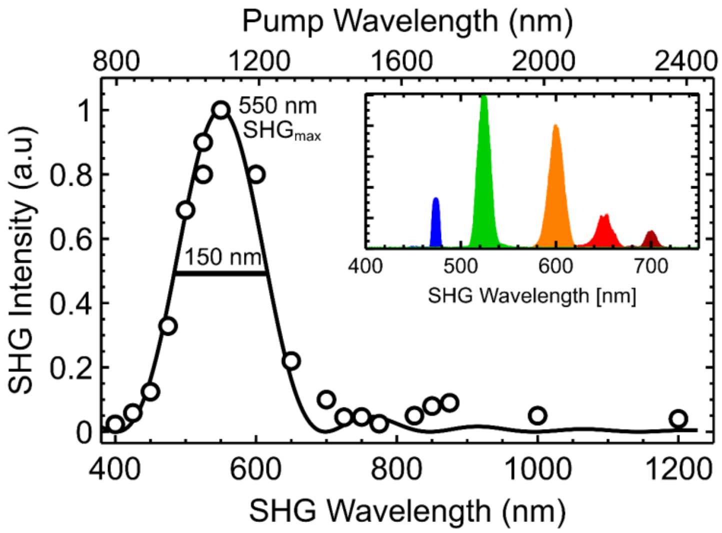

Figure S6. Normalized second harmonic generation from a Kainate crystal using a range of ultrashort pump wavelengths from 1000 – 1500 *nm*, where the SH signal is optimized through angular rotation of the crystal relative to the incident pumping pulse.

S8. Full Nonlinear Tensor Components

The full nonlinear quadratic tensor of Kainate was calculated at 1240 nm. The following table is the summarized data as extracted from MOPAC in [pm/V^{-1}]

	xx	*xy*	*yy*	*xz*	*yz*	*zz*
x	***-0.44996***	***1.932783***	***-1.52986***	***-0.50723***	***-1.10445***	***0.695393***
y	***1.840746***	***-1.54009***	***7.68***	***-1.07786***	***-2.00437***	***1.493049***
z	***-0.57268***	***-1.01241***	***-2.11481***	***0.603356***	***1.390786***	***-1.24762***

References

(1) He, Z.; Cremer, D. Sixth-Order Many-Body Perturbation Theory. I. Basic Theory and Derivation of the Energy Formula. *Int. J. Quantum Chem.* **1996**, *59* (1), 15–29. https://doi.org/10.1002/(sici)1097-461x(1996)59:1<15::aid-qua3>3.0.co;2-#.

(2) Bogdanov, A. A.; Shalin, A. S.; Ginzburg, P. Optical Forces in Nanorod Metamaterial. *Sci. Rep.* **2015**, *5*, 15846. https://doi.org/10.1038/srep15846.

(3) Ginzburg, P.; Krasavin, A. V.; Shalin, A. S.; Belov, P. A.; Kivshar, Y. S.; Zayats, A. V. Classical and Quantum Opto-Mechanics with Plasmonics and Metamaterials. In *CLEO: 2014*; OSA: Washington, D.C., 2014; p FTu3C.2. https://doi.org/10.1364/CLEO_QELS.2014.FTu3C.2.

(4) Concerning, R.; Law, T. H. E. C. Remarks Concerning the Clausius-Mossotti Law. **1929**, *49* (1850).
